# The Application of *Lamiaceae* Lindl. Promotes Aroma Compounds Formation, Sensory Properties, and Antioxidant Activity of Oat and Buckwheat-Based Cookies

**DOI:** 10.3390/molecules25235626

**Published:** 2020-11-29

**Authors:** Małgorzata Starowicz, Ewa Lelujka, Ewa Ciska, Grzegorz Lamparski, Tomasz Sawicki, Małgorzata Wronkowska

**Affiliations:** 1 Department of Chemistry and Biodynamics of Food, Institute of Animal Reproduction and Food Research of Polish Academy of Sciences, 10-784 Olsztyn, Poland; e.ciska@pan.olsztyn.pl (E.C.); m.wronkowska@pan.olsztyn.pl (M.W.); 2 Faculty of Biology and Biotechnology, University of Warmia and Mazury, 10-719 Olsztyn, Poland; ewalelujka@wp.pl; 3 Sensory Laboratory, Institute of Animal Reproduction and Food Research of Polish Academy of Sciences, 10-748 Olsztyn, Poland; g.lamparski@pan.olsztyn.pl; 4 Department of Human Nutrition, Faculty of Food Sciences, University of Warmia and Mazury, 10-718 Olsztyn, Poland; tomasz.sawicki@uwm.edu.pl

**Keywords:** bakery products, functional properties, HS-SPME-GC/MS, *Lamiaceae* herbs, sensory analysis, volatiles

## Abstract

Aroma plays an important role in designing innovative functional foods. This study aimed to study the influence of incorporating herbs from the *Lamiaceae* family (sage, mint, rosemary, oregano, thyme) on aroma compound formation and sensory properties in oat-buckwheat products. DPPH, FRAP and PCL have been used to describe possible antioxidant activity changes and reduce power of cookies after *Lamiaceae* Lindl. addition. The volatiles analysis by HS-SPME-GC/MS, has shown that *Lamiaceae* addition significantly influences the volatiles composition (29 molecules) with a predominance of molecules with a positive sensorial impression. Cookies elaborated with herbs were characterized by a greater share of monoterpenes (e.g., limonene, eucalyptol), in the volatile profile than in control cookies. These compounds’ occurrence was closely correlated with the appearance of herbal odor and taste among sensory attributes in cookies with herbs addition. In contrast, a decrease of negative oil aroma and the bitter aftertaste was noted by a sensory panel. Moreover, in cookies of mint and rosemary, hexanal share decreased about 13 and 9.7-times, respectively. Considering all presented experiments, rosemary addition was the most effective in forming a positive aroma profile with high sensory acceptance and increased functional properties.

## 1. Introduction

One of the main trends in the food industry nowadays is the development of functional foods. There is no strict definition of functional food. Therefore, functional food is a term used to describe “food products which have been enriched with natural substances/components with a specific physiological preventive and/or health-promoting effect” [1]. Functional food can have a positive effect on the human body, which can be caused, among other things, by the content of antioxidants. Therefore, it is crucial to enrich the diet with products containing high levels of antioxidants, such as grains, vegetables, fruits, spices, and/or herbs.

Oat, grouped as an ancient cereal, is a high-value grain, used in feed, but more often also in food production. In recent years, oat consumption has increased to about 5%, while in the European Union, over 9% is used for food purposes [2]. Oat is characterized by high-fat content (4–9%) and lower carbohydrate content than other types of cereals. It should be emphasized that oat and its products are a good source of B vitamins, fat-soluble vitamins (A, D, E, K), as well as compounds with high antioxidant properties (e.g., specific for oat polyphenols as aventramides) [3]. Another example of a valuable ancient crop is buckwheat. Buckwheat contains a large amount of essential polyunsaturated fatty acids, such as linoleic acid (18:2), vitamins (B, C, and E), minerals, and antioxidants (flavonoids and phenolic acids, e.g., rutin and quercetin) [4]. Moreover, to the group of bioactive compounds-rich plant material, herbs of *Lamiaceae* Lindl. can be included. Antioxidant, antimicrobial, and anti-inflammatory properties characterize the representatives of the *Lamiaceae* family. Each herb contains a unique, complex mixture of bioactive compounds [5]. They include secondary metabolites such as phenolic compounds, tannins, quinones, lignans, terpenoids, and flavonoids [6]. Herbs of *Lamiaceae* Lindl. are rich in essential oils, and therefore they can significantly influence the taste and aroma of the final product [7].

The high nutritional value of functional food should be linked with the high sensorial properties of designed food. Sensory acceptance is an essential aspect of the consumers’ perspective. The primary sensory features are taste and aroma. The aroma of baked goods is a composition of various volatile compounds that may come from raw materials or may arise during thermal processing, in this case, via baking (Maillard reaction compounds) [8]. The main aroma molecules identified in bakery products are aldehydes, alcohols, ketones, pyrazine, and furan derivatives [9]. In our previous study [9], we found that flavonoid, 3β-d-rutinoside, to cookies recipe increases favourable aroma compounds formation; however, it doesn’t influence sensory acceptance. We decided to add a naturally rich source of antioxidants such as herbs to the cookies formula and checked differences between volatiles and sensory aspects.

The study aimed to investigate the profile of volatile compounds and sensory attributes in cookies prepared from oat, buckwheat flours, and *Lamiaceae* Lindl. herbs (sage, mint, rosemary, oregano, thyme). Moreover, the changes in volatile compounds formation will be compared with the antioxidant activity variation and reducing cookies’ potential. To achieve this goal, the volatile compounds profile of oat-buckwheat cookies was obtained by HS-SPME/GC–MS. In addition sensory analysis, antioxidant activity and reducing potential (DPPH, photochemiluminescence-PCL and FRAP assays) were determined in oat-buckwheat cookies enriched with *Lamiaceae* Lindl.

## 2. Results

### 2.1. Volatile Compounds in Control Cookies and Cookies with Lamiacae Herbs Addition

Based on of the HS-SPME-GC/MS analysis, volatile compounds were identified in the control sample (without herbs addition) and in the samples fortified with sage, mint, rosemary, oregano, and thyme (Table 1). The results were presented as a percentage of the total peak area of each sample. According to data collected, aroma description and linear retention indices were assigned to each volatile compound, for polar column DB-WAX, literature, and databases [9,10,11,12]. Their specific mass spectra also identified molecules.

Monoterpenes (nine compounds), aldehydes (five), and alcohols (four) are the main volatile molecules identified in the samples. The representatives of other chemical classes as furan and pyrazine derivatives (two furans and two pyrazines), ketones (two compounds), thiazoles (one), and single representatives of lactones, sesquiterpenes, and acids were also identified. In total, 29 volatiles were identified, whereas 14 in the control sample and 12 to 21 compounds in samples fortified with herbs (Table 1). In the control sample, hexanal had the most significant contribution to the volatile composition. The relative abundance of hexanal decreased in samples with herbs addition, 2-times in cookies with sage to 12-times in cookies with mint. Hexanal has a green and grassy note, and it has a negative effect on human health [13]. In this aspect, it means that mint and rosemary are the most effective herbs in hexanal inhibition.

Control samples were also characterized by high abundance of benzaldehyde (burnt sugar odor), 2,5-dimethylpyrazine and 2-methylpyrazine (as a sum of 22.59% of the total volatile amount). Pyrazines possess characteristic positive cocoa, roasted, and nutty aroma, respectively. The nutty aroma can also be linked to the presence of 4-methylthiazole. Cookies prepared with sage were characterized by the most abundant, γ-butyrolactone with sweet and caramel aroma (about 26.79% of aroma profile), and hexanal, it equaled 18.58%. Only in sage samples, carvacrol was not identified. In samples of cookies with sage, an increase abundance of 2,5-dimethylpyrazine and 2-methylpyrazine was significant.

2-Methylpyrazine with a pleasant nutty aroma, has significantly higher percent contribution in volatile profile in cookies with herbs addition, the highest in samples with mint, then rosemary and sage. However, 2,5-dimethylpyrazine (cocoa and roasted aroma) was not identified in all samples, in sage and oregano, it remains at 9.59 and 9.05% of contribution to aroma profile. Moreover, in cookies with herbs appeared salicylaldehyde with aroma typically described with a pungent and spicy note (except cookies with thyme). Therefore, in cookies with mint, 2-heptanal described as pungent and green aroma, constituted the greatest volatiles share (21.13%). This volatile chemical was only identified in mint cookies. The great contribution in mint cookies’ volatiles was significant in γ-butyrolactone and 2-methylpyrazine percent of volatile identified (10.59 and 10.76%, respectively). A significantly higher abundance of caproic acid (7.20% of peak areas) in comparison to other samples was found. The presence of caproic acid might increase pungent and rancid taste and aroma sensations by consumers. In mint samples, also some monoterpenes as eucalyptol, limonene, and carvacrol have been determined. They resulted in a lower relative share than other volatiles; however, they might significantly impact the overall acceptance of final products.

The larger number of 21 volatile compounds was identified in oat-buckwheat cookies with rosemary addition. A higher abundance of eucalyptol (28.21% of total peak area) was found in rosemary cookies. Therefore, monoterpenes were an important part of the aroma (46.71%), associated with herbal notes e.g., camphor, minty, and anise. Also, pyrazines constituted the largest part of the volatile composition in rosemary samples, 8.48% for 2-methylpyrazine and 7.43% for 2,5-dimethylpyrazine. One of the monoterpenes (31.61%) carvacrol migh be the main aroma-forming compound for cookies with oregano. The oregano cookies were also characterized by a large abundance of hexanal (12.4%). However, compared to the control sample hexanal presence was almost 3-times lower. Furfural alcohol was only identified in cookies fortified with oregano, wherein in this case, furans constituted at 10.18% of total volatiles. In cookies with thyme addition, the lowest number (12) of volatiles has been identified. A great amount of cymene (22.33%) and carvacrol (17.43%) was found out in these samples. This means that above 40% of aroma is determined by monoterpenes. Important aroma components of thyme cookies were ketones (2-heptanone and 6-methyl-5-hepten-2-on) and aldehydes (hexanal and benzaldehyde), 19.38 and 17.31%, respectively. Ketones are responsible for shaping banana-like and citrus-like, fruity odor, whereas green, grassy, and burnt sugar aroma is characteristic for aldehydes.

### 2.2. Sensory Profile of Control Cookies and Cookies with Lamiacae Herbs Addition

**Figure 1:**
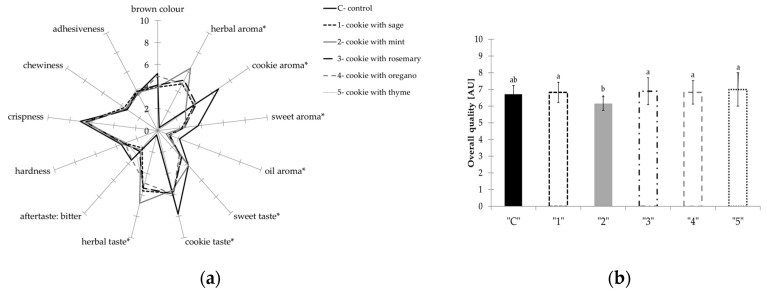
Spider diagram presenting the effect of herbs addition on QDA parameters (**a**) and overall quality of oat-buckwheat cookies (**b**). “*” in Figure 1a marked that results were statistically significant differences indicated by *p* ≤ 0.05. a, b- values marked with the same letter are not statistically significant in Figure 1b (Tukey test, *p* ≤ 0.05).

In the examined cookies samples, 13 individual quality features were taken into account, including one attribute of appearance (brown color), four aroma characteristics (herbal, cookie, sweet, oily), four taste characteristics (sweet, cookie, herbal, aftertaste defined as bitter), and four texture determinants (hardness, crispness, chewiness, and adhesiveness in mouth). An overall score was also determined for all samples, which summarizes the sensory quality based on all the individual characteristics considered: appearance, aroma, taste and texture. The discriminants describing the quality characteristics of the tested samples along with their definitions and boundary terms of the scale are presented in Table 2, and the average results from two independent cookie evaluation sessions are presented as spider diagram Figure 1a. The obtained results indicate that the tested samples differed statistically significantly in terms of aroma and taste. There were no statistically significant differences in the texture of cookies (Figure 1a) and in the overall evaluation (Figure 1b).

By analyzing the features describing the aroma and taste of the analyzed cookie samples with the addition of mint, characterized by the highest value of herbal aroma (6.4 c.u.) and taste (6.8 c.u.). While the control sample was characterized mainly by the highest value of cookie (6.7 c.u.) and sweet odors (3.7 c.u.), and cookie taste (7.8 c.u.). The control sample was also the sweetest, determined as 4.2 on the the10-point scale (c.u.). There was a decrease in the value of the aroma and cookie taste in cookies with herbs addition, in favor of the herbal aroma and taste.

### 2.3. Antioxidant Activity and Reducing Potential of Cookies

The antioxidant activity of oat-buckwheat cookies extracts with the herbs’ addition was determined using two methods, such as DPPH and PCL. Based on the DPPH assays, the highest antioxidant activity was recorded in the extract of cookies enriched with mint (4.23 mmol Trolox/g; Table 2), indicating an increased influence of polyphenolic compounds derived from the mint on shaping the antioxidant activity of the analyzed cookies. In addition, the extracts of cookies with the rosemary addition (3.83 mmol Trolox/g) and sage (3.48 mmol Trolox/g) were characterized as equally high antioxidant potential. The differences between these cookies were not statistically significant. Two times lower radical scavenging ability against DPPH radicals was obtained in the extracts of cookies with oregano (1.90 mmol Trolox/g) compared to the rosemary cookies. On the other hand, the lowest values of the antioxidant potential determined by DPPH assay were recorded in the extracts of cookies with the addition of thyme and the control cookies (without herbs), 0.09 and 0.06 mmol of Trolox/g of the sample, respectively.

The PCL method was used to characterize oat-buckwheat cookies’ ability to scavenge superoxide anion radical. The obtained results are also presented in Table 2. The ACW (water-soluble antioxidant compounds) values were in the following order: cookies with the addition of mint > sage > oregano > rosemary > thyme. The highest antioxidant activity was determined in the extract of cookies using mint (9.32 µmol Trolox/mL sample). This result was three times higher compared to the control cookie extract. The lowest antioxidant activity among the extracts of cookies with the addition of herbs was found in the extract of cookies with thyme. Its antioxidant activity value was twice as high as that determined for the control cookies. Furthermore, ACL (lipid-soluble antioxidant compounds) values were obtained as follows: cookie with sage> oregano> rosemary> mint> thyme. The highest value was recorded in the extract of cookies with the addition of sage (42.92 µmol Trolox/mL of the sample). This result was four times higher than the data determined for the control and two times higher than the value obtained for the extract of cookies with the addition of oregano. A similar antioxidant activity level characterized the extracts of cookies with the mint and rosemary. In contrast, the lowest value of the antioxidant activity was recorded for the extract of cookies with thyme (7.08 µmol Trolox/mL of the sample).

The highest value of the reducing capacity analyzed by the FRAP method was recorded for the extract of cookies with rosemary (2.67 mmol Trolox/g of sample) (Table 2). The cookie extract with the addition of mint also was characterized by an equally high result. Moreover, the cookie extracts with the addition of sage, thyme, and oregano had a reducing capacity at a similar level. The reducing potential of sage cookie extracts was 22% lower than rosemary, followed by 19% for thyme and 18% for oregano cookies. The low reducing potential was noted for the control cookies (1.33 mmol Trolox/g of sample). This value was two times lower compared to the rosemary cookie extract.

## 3. Discussion

### 3.1. Volatiles Identified in Analysed Cookies with Herbs Addition

Lasekan and Lasekan [14] showed that alcohols, aldehydes, ketones, benzene derivatives and terpenoids dominate in the volatile profile of buckwheat products, which is confirmed by the results of the GC/ MS analysis of the control cookie, noted in Table 1. Among the 29 volatile compounds identified in the cookies, there were molecules such as nonanal and hexanal. Aoki and Koizumi [15] showed that they are aromatic compounds derived from buckwheat but with low odour thresholds. On the other hand, Sides et al. [16] identified these aldehydes in oat samples. It could mean that nonanal and hexanal are originated from used both cereals/ flours. Chemical compounds belonging to pyrazines were also detected in the cookies: 2-methyl- and 2,5-dimethylpyrazine. These compounds were identified in control, cookies with the addition of sage, mint, rosemary and oregano. Based on the results, it was found that herbal supplementation to oat-buckwheat cakes increased the total abundance of pyrazines. Pyrazines are heterocyclic and aromatic compounds with characteristic pleasant nutty odour notes. They might be important factors influencing the aroma and taste of thermally treated food, such as cocoa, coffee, nuts, popcorn, crisps or bread [17]. Starowicz et al. [9] confirmed that pyrazines could be found in cookies, and pyrazines content can be modified by antioxidant enrichment. In this case, herbs of *Lamiaceae* Lindl. might be a rich natural source of antioxidants and promote pyrazines formation in cookies with their fortification. The effectiveness of pyrazines formation in sweet cookies is also related to sugar usage in their recipe. As it was presented by Koutsidis et al. [18], glucose accelerates pyrazine formation in the model system.

Apart from pyrazines, also some furan derivatives as furfural and furfuryl alcohol were found in this study. Furan derivatives are a part of research dedicated to baby food because of their “carcinogenic and cytotoxic activity on animals and harmful effects on human health” [19]. Therefore, the presence of furans is required to be controlled. However, Srivastava et al. [20] pointed out that furfural content increases the positive aroma of cereal products. Also, hexanal could have some negative impacts on human health. It was found that hexanal irritates the mucous membranes of the human respiratory and digestive system [13]. It turned out that supplementing cookies with herbs reduces this compound’s content, which is a beneficial effect. The addition of mint to oat-buckwheat cookies reduced this compound by 91%, rosemary by 81%, thyme by 81%, and sage by 52% compared to the control.

An important role in aroma formation of oat-buckwheat cookies fortified with *Lamiaceae* herbs plays monoterpenes (limonene, eucalyptol, cymene, thujone, menthol, terpineol, borneol, thymol, and carvacrol). Monoterpenes are the main components of essential oils, and therefore they are most abundant in herbs tissues [21]. For example, Yadegarinia et al. [22] detected high content of monoterpenes in peppermint (*Mentha piperita* L.) using GC/MS analysis. Therefore, in cookies with oregano and thyme, high content of monoterpene- carvacrol was determined. This compound, except for aroma-shaping properties, has many functional values, including antimicrobial, and anticancer [23]. Carvacrol was also characterized by high antioxidant activity [23]. Furthermore, monoterpene as eucalyptol was found in cookies with rosemary. According to these findings, it can be assumed that herbs are a main source of monoterpenes in studied products.

Therefore, there are two crucial aspects of aroma formation in studied cookies. One of them is thermal processing e.g., baking. It is a dynamic process involving heat that leads to physical and chemical changes in products. High temperatures trigger reactions that produce large amounts of compounds. Many of them contribute to improving the quality of taste, aroma and colour [24]. Many scientific articles deal with the subject of the Maillard reaction and indicate the products of this reaction as essential food components. For this reason, the study of the effects of this reaction-both positive (e.g., pyrazines) and negative (e.g., furan derivatives) is an important topic in today’s research [25]. The essential aspect of functional food designing is to improve sensory attributes and achieve high consumer acceptance.

### 3.2. Sensory Profile of Cookies after Herbs Addition

The sensory evaluation showed that the addition of sage, mint, rosemary, oregano, or thyme to oat-buckwheat cookies improves baking goods’ sensory quality. High sensory quality (apart from the price and nutritional value) greatly influences consumers’ choice of product. The result of the present study confirm those obtained by Przygodzka [26]. Researchers enriched rye-buckwheat cakes with cloves, nutmeg, allspice, cinnamon, vanilla, and a mixture of spices for ginger cakes. Fortifying cookies with these spices also improved the overall baking quality and increased their sensory properties.

The beneficial effect of enriching baked goods with herbs from the *Lamiaceae* family reduces the subsequent bitter taste in case of the reduction of herbal flavour. This is primarily important from the point of view of consumers. Consumers do not prefer intense bitterness in food and choose its low or moderate-intensity [27]. The interesting solution for consumers and food producers was proposed to reduce the bitterness in buckwheat-based foods by Suzuki et al. [28]. Suzuki et al. [28] attempted to develop a new variety of buckwheat, the *‘Manten-Kairi’* Tartary buckwheat, which would be devoid of bitterness. The flour obtained from *‘Manten-Kairi’* was compared with that obtained from *‘Hokkai T8’* buckwheat; for this purpose, a sensory analysis was carried out. The panelists participating in this study did not report a bitter taste in a new variety. Therefore, this variety of Tartary buckwheat could increase this pseudo-cereal consumption in the future and become a promising material for preparing of functional products rich in buckwheat.

**Figure 2:**
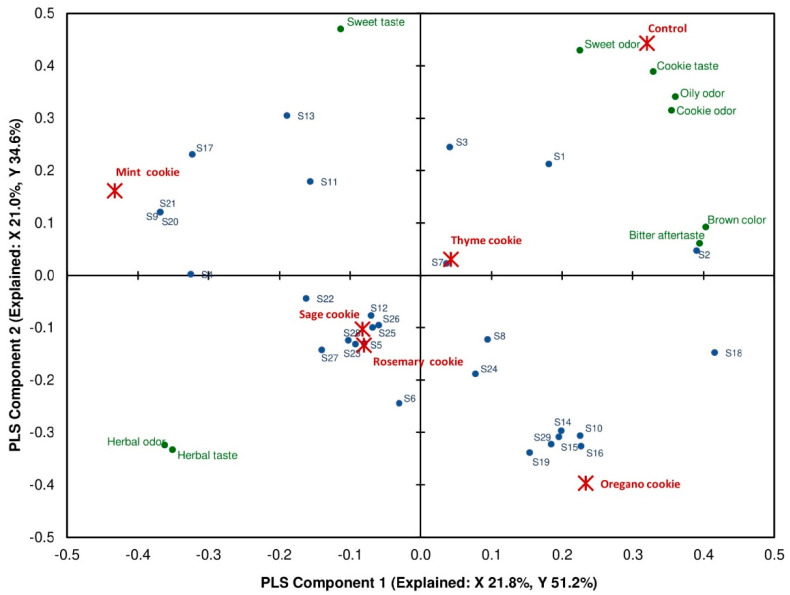
PLS correlation analysis of samples, between aroma compounds and sensory attributes. S1–S29 number of compounds identified in the analyzed samples of control, and cookies with herbs addition: sage, mint, rosemary, oregano, and thyme.

Figure 2 shows a PLS analysis between aroma compounds and sensory attributes (aroma and taste) of analyzed samples. Control cookies were positively associated with the various sensory characters (sweet, oily and cookie odor, and cookie and sweet taste), which may be positively influenced by the presence of 1-heksanol and 2-heptanon. On the other hand, the cookies with the herbs fortification were positively correlated with herbal odor and taste. Cookies with sage were highly positively associated with the concentration of eight volatile compounds but mostly with thymol, terpineol, borneol, thujone, caproic acid, eucalyptol and γ-butyrolactone. In addition, sage cookies were also correlated with 2-methylpyrazine, which possesses a pleasant nutty aroma. Furthermore, the mint cookies were positively associated with the eight volatile compounds. The highest correlation was achieved in the case of 2-heptanal, 1-octanol, and β-caryophyllene, whereas correlation with γ-butyrolactone, limonene, 2-ethylhexanol, nonanal, and 6-methyl-5-hepten-2-on was positive but lower. Moreover, the high positive correlation of 2-heptanal in mint cookies is presented in sensorial analysis, lower overall acceptance of panelists’ mint cookies. In other samples of rosemary cookies, high correlations were noted for eight volatile molecules. They are the same compounds that were identified in sage cookies. Two other compounds as 4-methylthiazole and salicylaldehyde are positively associated with sensorial attributes of cookies with rosemary addition. The nine aroma compounds presented a positive correlation with sensorial descriptors of aroma and taste in samples with oregano. These compounds are: 4-methylthiazole, 2,5-dimethylpyrazine, furfural alcohol, acetic acid, furfural, benzaldehyde, linalool, salicylaldehyde, and carvacrol). Besides them, also positive correlation was noted for 2-methylpyrazine. Last sample of cookies with thyme noted that the thyme cookies were very highly correlated with only one detected compound, cymene. A lower correlation was presented between thyme cookies and the presence of hexanal, 1-hexanol, and 2-heptanone. Then, interesting observations were noted that hexanal with green and grassy aroma was highly correlated with a bitter aftertaste. Thyme and control cookies are the most similar samples in aspects of sensory features, it could mean that the addition of thyme did not a strongly affect oat-buckwheat cookie acceptance.

According to these observations, it could be stated that the sensory attributes and volatile compounds profile of analyzed cookies were significantly affected by the type of added herbs. The unique sensory descriptors of studied cookies were responsible for the superimposed and synergistic effects of individual volatiles.

### 3.3. Changes in Antioxidant Activity and Reducing Power after Herbs Addition

This analysis confirms the positive effect of the addition of family Lamiaceae herbs to the oat-buckwheat cookies on the final product’s antioxidant activity. May be due to the presence of phenolic compounds in herbs, e.g., rosmarinic acid. Chen and Ho [29] showed that it is an essential compound found in herbs from the Lamiaceae family, at the same time characterized by a high DPPH radical scavenging capacity. Cookies without the addition of herbs (control) were characterized a low antioxidant potential, although oats and buckwheat have a high ability to scavenge radicals [30]. In recent years, a lot of research has been done on the enrichment of baked products. Much research has focused on the fortification of wheat bread with leaves, stems, roots, and seeds of herbs, rich in micronutrients, antioxidants, essential oils, and fiber [31]. Das et al. [31] analyzed the antioxidant properties of wheat bread enriched with powdered coriander leaves. The researchers noted the maximum increase of antioxidant activity after fortification of bread with coriander in the DPPH method by 187% (7% supplementation) and in the FRAP method by 108% (7% supplementation), compared to the control. Our study’s results also showed a significant increase in the DPPH method’s antioxidant activity than in the FRAP method. Table 2 observed the maximum increase in antioxidant potential in the extract of cookies with mint. This result was 25 times higher than the result obteined by the researchers mentioned above, who used powdered coriander as plant material. Proves the higher antioxidant potential of mint than coriander and its significant influence on shaping the final product’s antioxidant potential. The highest reducing capacity was determined for the extract of rosemary cookies, which was similar to the result obtained by Das et al. [31]. In another study, Wang et al. [32] used the addition of powdered celery of two varieties ‘Jinnan Shiquin’ and ‘Ventura’ for the baking of wheat bread. In the cited study, the antioxidant activity was also measured by two methods (DPPH and FRAP). The addition of powdered celery added to baked products increased the antioxidant activity determined by DPPH assay by approximately 44.9–67.2% for ‘Jinnan Shiquin’and 39.8–65.3% for the ‘Ventura’ varieties. Our study indicates that the addition of herbs from the Lamiaceae family to oat-buckwheat cookies also increased baked products’ antioxidant activity. The results were over 40 times higher than the researchers mentioned above’ results, which confirms that a very high antioxidant potential characterizes herbs from the Lamiaceae family. Moreover, bread fortified by celery was also marked by the increase of reducing capacity by about 23.5–213.3% for ‘Jinnan Shiqin’ and 27.6–196.0% for ‘Ventura’ [32]. In the present study, the supplementation of cookies with herbs from Lamiaceae family, increase the reducing potential by 55.6–100.8%.

Another example of enriching baked products is adding fruit, fruit pomace, or fruit powders. Tańska et al. [33] added rosehip, blueberry, rowan, and blackcurrant fruit pomace to traditional wheat cookies. Such fruit juice production residues are a good source of bioactive compounds, polyphenols, vitamins, provitamins, fatty acids, and dietary fiber. Moreover, in this study, a 4-fold increase in the antioxidant potential determined by DPPH method was noted in the cookies supplemented with pomaces. In our study, the addition of herbs from the Lamiaceae family increased the DPPH radical scavenging activity more than 40 times compared to the control. This means that the herbs used in this study are a better antioxidant source than the fruits mentioned above. Moreover, the work of Tańska et al. [33] also confirms a strong correlation between the antioxidant activity of fruit pomace and its total phenolic content. A significant increase in the antioxidant potential after supplementing cookies with herbs from the Lamiaceae family in our study may prove the high content of phenols in the used herbs.

Based on the PCL analysis, an increase in the research product’s antioxidant activity was noted after supplementation with herbs from the *Lamiaceae* family, and a more significant share of lipid-soluble substances than water-soluble substances was determined in each trial. In the extract of cookies with sage, the ACL value was six times higher than that of the ACW. This is because herbs from the *Lamiaceae* family are rich in essential oils [34]. Higher ACL values than ACW were also noted by Szawara-Nowak et al. [35]. The results concluded that the higher the percent of substitution of wheat flour with another type of flour (characterized by high antioxidant activity), the higher the ACL values compared to ACW. In the cited study, the researchers replaced the wheat flour for baking dark wheat bread with buckwheat flour or buckwheat flour obtained from buckwheat and added them in the amount of 10, 20, 30, and 50%, which resulted in a significant increase in the antioxidant activity of the baked product.

## 4. Materials and Methods

### 4.1. Preparation of Experimental Oat-Buckwheat Cookies: Control and with Lamiacae Herbs

**Figure 3:**
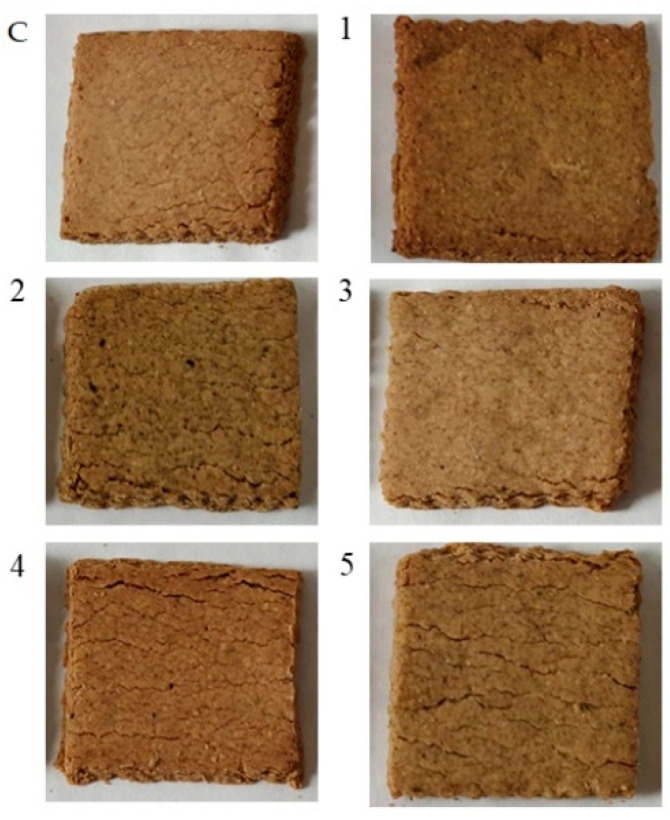
Oat-buckwheat cookies (C- control, without herb addition) and with the fortification of herbs: 1- sage, 2- mint, 3- rosemary, 4- oregano, 5- thyme.

The material used for the study was experimental oat- buckwheat cookies (Figure 3). The control cookies were prepared according to the recipe: 1 egg, 400 g of buckwheat flour, 400 g of oat flour, 180 g of oil, 100 g of sugar, 12 g of baking powder, 160 g of water. Therefore to cookies with *Lamiacae* herbs, 16 g of herb was additionally added. The amount of added herbs were established according to preliminary sensory studies (data not shown). The whole ingredients were mixed (VC750, Sonics & Materials, Newtown, CT, USA), then the dough was hand-made, rolled, cut into 40 pieces of 6 × 6 cm and baked (DC-21, SVEBA DAHLEN AB, Fristad, Sweden) at 200 °C for 25 min. After baking, the biscuits were allowed to cool to room temperature. 30 cookies of each type were used for sensory analysis. The samples were selected without mechanical damage. The remaining part was ground (WZ-1, Zakład Badawczy Przemysłu Piekarskiego, Bydgoszcz, Poland), closed in paper bags and freeze-dried. The lyophilizates were then ground and stored in a freezer (at −80 °C) until the appropriate extracts were prepared.

Mint (*Mentha* L.), thyme (*Thymus vulgaris* L.), rosemary (*Rosmarinus officinalis* L.), sage (*Salvia officinalis* L.), and oregano (*Origanum vulgare* L.) were herbs used in the preparation of experimental cookies. The herbs were purchased at the online store www.ziolowyzakatek.sklep.pl (Grodzisk, Podlaskie Voivodeship, Poland). Before use, the herbs were ground into a powder, which was then directly used in oat-buckwheat cookies.

### 4.2. Volatile Compounds Determination in Oat-Buckwheat Cookies by HS-SPME-GC/MS

The GC-MS method developed by Starowicz et al. [9] was used to determine the volatile compounds in oat-buckwheat cookies. Extracts Therefore, 2 g of freeze-dried cookie were weighed into a 20-mL vial and 2.5 mL of 20% sodium chloride solution was added. The vials were then placed on a shaker (Eppendorf, Hamburg, Germany). Samples were shaken and heated (40 °C, 45 min, 650 RPM), and then the volatiles were allowed to absorb onto the SPME fiber for 15 min at 40 °C (without shaking). A 50/30 µm stable DVB/CAR/PDMS fiber (Supelco, Bellefonte, PA, USA) was used. The injection was done manually. Thus, the SPME fiber was introduced into the chromatograph injection port, where the analytes were desorbed (250 °C, 5 min) and transferred on a capillary column (DB-WAX, 30 m, 0.25 mm × 0.50 µm). The analyses were performed on a gas chromatograph (Agilent Technologies 7890A GC system, Santa Clara, CA, USA) coupled to a mass spectrometer (Agilent Technologies 5975C VL MSD, Santa Clara, CA, USA). The temperature was initially set to 40 °C and held for 5 min. The temperature was then increased to 200 °C and held for 1 min. Finally, it was increased to 240 °C and held for 5 min. In the method, helium was used as the carrier gas with a flow rate of 1 mL min^−1^ in the splitless mode. The flow was kept constant.

The analyses were carried out in triplicate. The compounds were identified by comparing the obtained linear retention indices, retention times and mass spectra with the Wiley Registry 7th Edition Mass Spectral Library (Wiley and Sons Inc., Weinheim, Germany) and the National Institute Standards and Technology (NIST) 2005 Mass Spectral Library. Linear retention indices (LRIs) were calculated using C_6_-C_30_ n-alkenes mix (Sigma-Aldrich, St. Louis, MO, USA). The results were presented as the percentage of total peak area of the individual compounds identified in the database.

### 4.3. Sensory Analysis of Oat-Buckwheat Cookies Enriched with Herbs

The sensory evaluation was performed using the quantitative descriptive analysis (QDA) method called sensory profiling (quantitative and qualitative method). Following the method procedure [36], a list of characteristics of the tested products, the distinguishing features was prepared. The discriminants were defined in order to be equally understood by the team members. Then the intensity of each of these features was assessed on a continuous scale (corresponding to 10 conventional units—c.u.), with boundary terms described (Table 3). The boundary terms of the scale for the aroma and taste discriminants were: 0- “non-intense”–10- “very intense”. The panel estimated the overall quality of the samples, which summarizes the assessed products’ sensory quality based on all unit characteristics included in the research. The overall quality was scored from 0 to 10, whereas 0 described low quality and 10- high quality.

The evaluation was carried out by a 7-person team—selected, trained and monitored in accordance with PN-EN ISO 8586: 2014 [37], with appropriate methodological preparation and experience in sensory profiling of various products. The assessments were carried out in the Sensory Laboratory of the Institute of Animal Reproduction and Food Research of the Polish Academy of Sciences in Olsztyn, meeting the requirements of ISO 8589: 2010 [38].

The test samples were oat-buckwheat cookies: control (without the addition of herbs), and cookies with the addition of herbs: 1—sage, 2—mint, 3—rosemary, 4—oregano, 5—thyme. A cookie from a given batch was randomly selected for the evaluation. Each evaluator received 6 cookies for evaluation. The samples were sealed in disposable containers which were individually coded and presented to the panellists in a randomized order (different for each panellists) in two independent sessions. Mineral water was administered to the panellists as a neutralizing agent. The assessments were carried out on individual stands at room temperature.

### 4.4. Determination of Antioxidant Activity (DPPH, PCL) and Reducing Power (FRAP) of Cookies

About 200 mg of powdered cookies were extracted with 80% methanol according to methodology described by Przygodzka et al. [26]. Then extracted samples were stored in −80 °C before prior analysis of antioxidant and reducing capacity (DPPH, PCL, and FRAP assays).

Therefore, DPPH assay was established to measure antioxidants (extracted from cookies) scavenging ability against DPPH radicals [39]. The measurement were performed at a microplate reader (Tecan M1000 Infinite PRO) with the wavelength established at 517 nm. Results were presented as mmol Trolox per gram of sample. PCL method was used to measure ability of antioxidants from cookies to scavenge superoxide anion radicals (O_2_^−•^). The measurement was performed using PHOTOCHEM apparatus (Analytik Jena, Jena, Germany) according to protocols elaborated by Zieliński et al. [40]. The extracts of cookies were determined in two methodologies: for lipophilic antioxidants (PCL ACL) and hydrophilic (PCL ACW) methodology extracts of were expressed as µmol Trolox per gram of sample. The reducing power was determined using FRAP assay according to Horszwald and Andlauer [39]. The mixture’s absorbance was measured at 593 nm after 5 min reaction with microplate reader (M1000 Infinite PRO, Tecan, Männedorf, Switzerland). The FRAP method is based on the reduction of ferric ion by antioxidant compounds.

### 4.5. Statistical Analysis

The data are presented as mean values and standard deviations of triplicate measurement. The differences between samples were analysed by a one-way ANOVA with Tukey’s multiple comparison test (*p* < 0.05) using STATISTICA 7.0 (StatSoft Inc., Tulsa, OK, USA).

The computerized sensory program FIZZ (Biosystemes, Counternon, France) was used to prepare and perform the sensory evaluation, and then to compile individual results and graphical presentation. The results are presented as the mean of the two sessions. For the statistical analysis of the sensory results, the LSD test and the one-way analysis of variance (ANOVA) were used at the significance level *p* < 0.05 (Addinsoft, XLStat ver. 19.01, New York, NY, USA).

Moreover, partial least squares (PLS) method was used to investigated the relationship between cookies, aroma compounds and sensory attributes. The software used for PLS was R version 4.0.2 (R Foundation for Statistical Computing, Vienna, Austria), with the use of appropriate packages.

## 5. Conclusions

The sensory quality of innovative bakery products can be increased by adding natural aromatic ingredients such as herbs. Therefore, in this study, 29 volatile compounds have been identified in analyzed oat-buckwheat cookies with *Lamiacae* Lindl. According to the obtained results, an important role in the aroma formation of cookies plays monoterpenes. A positive effect on lower relative abundance of hexanal and higher in case of pyrazines was noted after herbs addition to control cookie recipe. Moreover, antioxidant activity and reducing power increased significantly after sage, mint, rosemary, and oregano. However, considering the performed analysis, rosemary addition was the most effective in forming a positive aroma profile with high sensory acceptance and increased functional properties.

## Figures and Tables

**Table 1:** Determination of volatile compounds in samples of cookies: C- control (without herbs) and 1- cookie with sage, 2- mint, 3- rosemary, 4- oregano, and 5- thyme.

No.	Compound	Chemical Family	Aroma Description	LRIs _exp._	LRIs _lit._	C- control	1	2	3	4	5
						[%] of Total Peak Area					
1	1-hexanol	Alcohols	fresh, fruity	888	863	3.65 ± 0.03 ^a^	1.84 ± 0.03 ^b^	-	1.32 ± 0.02 ^b^	-	-
2	hexanal	Aldehydes	green, grassy	1078	1098	38.26 ± 1.35 ^a^	18.58 ± 0.90 ^b^	2.93 ± 0.01 ^f^	3.94 ± 0.07 ^e^	12.64 ± 0.10 ^c^	8.20 ± 0.10 ^d^
3	2-heptanon	Ketones	banana-like	1174	1187	6.71 ± 0.5 ^b^	-	2.93 ± 0.05 ^d^	3.94 ± 0.05 ^c^	-	8.07 ± 0.05 ^a^
4	limonene	Monoterpenes	citrus	1180	1188	-	-	2.78 ± 0.02 ^a^	1.93 ± 0.01 ^b^	-	-
5	eucalyptol	Monoterpenes	herbal	1205	1214	-	-	4.27 ± 0.01 ^b^	28.21 ± 0.14 ^a^	2.63 ± 0.02 ^c^	-
6	2-methylpyrazine	Pyrazines	nutty	1236	1251	6.93 ± 0.02 ^d^	8.18 ± 0.70 ^b^	10.76 ± 0.09 ^a^	8.48 ± 0.04 ^b^	7.51 ± 0.05 ^c^	-
7	cymene	Monoterpenes	terpenic	1277	1281	-	-	-	-	-	22.33 ± 0.08
8	4-methylthiazole	Thiazoles	nutty	1280	1279	3.97 ± 0.02 ^b^	2.38 ± 0.05 ^c^	3.43 ± 0.03 ^b^	2.85 ± 0.02 ^c^	1.97 ± 0.02 ^d^	4.79 ± 0.01 ^a^
9	2-heptanal	Aldehydes	pungent, green	1319	1323	-	-	21.13 ± 0.22	-	-	-
10	2,5-dimethylpyrazine	Pyrazines	cocoa, roasted	1323	1320	7.09 ± 0.08 ^b^	9.59 ± 0.12 ^a^	-	7.43 ± 0.05 ^b^	9.05 ± 0.09 ^a^	-
11	6-methyl-5-hepten-2-on	Ketones	citrus-like, fruity	1375	1365	4.36 ± 0.01 ^c^	-	5.92 ± 0.08 ^b^	4.07 ± 0.01 ^c^	-	11.31 ± 0.15 ^a^
12	thujone	Monoterpenes	thujonic, herbal	1435	1438	-	1.70 ± 0.01	-	-	-	-
13	nonanal	Aldehydes	aldehydic	1440	1437	1.62 ± 0.01 ^b^	-	2.36 ± 0.02 ^a^	-	-	-
14	furfural alcohol	Furans	almond, sweet	1457	1458	-	-	-	-	2.52 ± 0.06	-
15	acetic acid	Acids	sour, pungent	1478	1477	-	3.88 ± 0.02 ^b^	-	-	6.26 ± 0.57 ^a^	-
16	furfural	Furans	sweet, fruity	1491	1485	1.80 ± 0.01 ^d^	2.32 ± 0.01 ^c^	-	2.28 ± 0.02 ^c^	7.66 ± 0.08 ^a^	3.07 ± 0.01 ^b^
17	2-ethylhexanol	Alcohols	fruity	1500	1504	3.74 ± 0.04 ^b^	3.36 ± 0.08 ^b^	7.86 ± 0.02 ^a^	0.99 ± 0.01 ^c^	-	-
18	benzaldehyde	Aldehydes	burnt sugar	1542	1525	8.57 ± 0.12 ^a^	6.21 ± 0.02 ^b^	-	4.36 ± 0.02 ^c^	4.04 ± 0.01 ^c^	9.61 ± 0.08 ^c^
19	linalool	Alcohols	floral, lemon-like	1550	1554	-	-	-	1.59 ± 0.01	2.17 ± 0.02 ^a^	-
20	octan-1ol	Alcohols	herbal	1563	1565	-	-	1.88 ± 0.01	-	-	-
21	β-caryophyllene	Sesquiterpenoids	woody	1627	1618	-	-	4.74 ± 0.05	-	-	-
22	γ-butyrolactone	Lactones	sweet, caramel	1639	1640	6.36 ± 0.13 ^c^	26.79 ± 0.15 ^a^	10.59 ± 0.08 ^b^	2.67 ± 0.03 ^d^	2.68 ± 0.02 ^d^	5.75 ± 0.01 ^c^
23	menthol	Monoterpenes	fresh, herbal	1622	1626	-	1.45 ± 0.01 ^b^	-	1.83 ± 0.01 ^a^	-	1.09 ± 0.02 ^c^
24	salicylaldehyde	Aldehydes	pungent, spicy	1668	1658	-	5.39 ± 0.05 ^c^	7.44 ± 0.02 ^a^	5.82 ± 0.05 ^c^	6.05 ± 0.02 ^b^	-
25	terpineol	Monoterpenes	minty, anise	1718	1719	-	-	-	4.22 ± 0.03	-	-
26	borneol	Monoterpenes	camphor, musty	1726	1721	-	-	-	4.85 ± 0.01 ^a^	-	3.28 ± 0.05 ^b^
27	caproic acid	Carboxylic acid	pungent, rancid	1866	1863	5.16 ± 0.02 ^b^	4.53 ± 0.04 ^c^	7.20 ± 0.05 ^a^	3.55 ± 0.02 ^c^	3.21 ± 0.08 ^d^	5.07 ± 0.08 ^b^
28	thymol	Monoterpenes	herbal	2155	2157	-	3.82 ± 0.03 ^a^	-	1.02 ± 0.01 ^b^	-	-
29	carvacrol	Monoterpenes	caraway	2180	2183	1.78 ± 0.01 ^d^	-	3.77 ± 0.02 ^c^	4.65 ± 0.05 ^c^	31.61 ± 0.02 ^a^	17.43 ± 0.15 ^b^

Values were presented as mean ± standard deviation. Values followed by different letters in the same column are significantly different (*p* < 0.05), as determined by the Tukey’s multiple comparisons test. “-” compound was not detected.

**Table 2:** Determination of antioxidant activity in extracts of oat-buckwheat cookies by DPPH, FRAP and PCL method (lipid-soluble antioxidant compounds- ACL and water-soluble antioxidant compounds- ACW).

	DPPH (mmol Trolox/g)	FRAP (mmol Trolox/g)	PCL ACW (µmol Trolox/mL)	PCL ACL (µmol Trolox/mL)
C-control	0.09 ± 0.00 ^d^	1.33 ± 0.11 ^c^	3.34 ± 0.32 ^c^	10.55 ± 0.07 ^d^
Cookies with:				
sage	3.48 ± 0.20 ^b^	2.07 ± 0.04 ^b^	7.16 ± 0.66 ^b^	42.92 ± 2.70 ^a^
mint	4.23 ± 0.04 ^a^	2.47± 0.12 ^a^	9.32 ± 0.70 ^a^	15.99 ± 0.31 ^c^
rosemary	3.83 ± 0.34 ^ab^	2.67 ± 0.10 ^a^	6.36 ± 0.49 ^b^	18.71 ± 0.71 ^c^
oregano	1.90 ± 0.26 ^c^	2.18 ± 0.13 ^b^	6.47 ± 0.58 ^b^	23.95 ± 0.59 ^b^
thyme	0.16 ± 0.00 ^d^	2.16 ± 0.08 ^b^	5.79 ± 0.19 ^b^	7.08 ± 0.27 ^e^

Values were presented as mean ± standard deviation. Values followed by different letters in the same column are significantly different (*p* < 0.05), as determined by the Tukey’s multiple comparisons test.

**Table 3:** Sensory attributes, their definitions and scale edges used in the QDA of experimental cookies.

	Attributes	Definition	Scale Edges
Color	brown color	color intensity (color pattern RAL 050 50 30-scale value 5)	light–dark
Aroma	herbal	aroma note characteristic for herbs like: sage, mint, rosemary, oregano and thyme	non-intensive–very intensive
	cookie	aroma typical for commercial muesli cookies	non-intensive–very intensive
	sweet	aroma note typical for sweet additives (e.g., vanilla sugar dissolved in water)	non-intensive–very intensive
	oil	characteristic aroma of commercially- available oil e.g., sunflower oil	non-intensive–very intensive
Taste	sweet	taste characteristic for saccharose solution	non-intensive–very intensive
	cookie	taste typical for commercial muesli cookies	non-intensive–very intensive
	herbal	taste associated with added herbs e.g., sage, mint, rosemary, oregano, and thyme	non-intensive–very intensive
	aftertaste: bitter	impression of bitterness that persists after swallowing the sample	non-intensive–very intensive
Texture (in mouth)	hardness	the force required to bite a sample placed between the front teeth	low–high
	crispness	the force required to crush the specimen within five bites with the molars	low–high
	chewiness	chewing the sample multiple times in order to prepare it for ingestion	low–high
	adhesiveness	degree of adhesion felt after 10 chews	low–high
overall acceptance		overall quality contain all attributes and their harmonization	low–high
